# The Isosteroid Alkaloid Imperialine from Bulbs of* Fritillaria cirrhosa* Mitigates Pulmonary Functional and Structural Impairment and Suppresses Inflammatory Response in a COPD-Like Rat Model

**DOI:** 10.1155/2016/4192483

**Published:** 2016-07-20

**Authors:** Dongdong Wang, Qingdan Du, Houcong Li, Shu Wang

**Affiliations:** ^1^Department of Pharmacognosy, West China College of Pharmacy, Sichuan University, No. 17, Duan 3, Renmin Nan Road, Chengdu 610041, China; ^2^Department of Pharmacognosy, Faculty of Life Sciences, University of Vienna, Althanstrasse 14, 1090 Vienna, Austria

## Abstract

Chronic obstructive pulmonary disease (COPD) is the third leading cause of death in the world. Present therapies for COPD have limited effect on reducing the progression of COPD and suppressing the inflammatory response in the lung. Bulbs of* Fritillaria cirrhosa* D. Don (BFC) have been used in many Asian countries for a long time to treat pulmonary diseases, such as cough, expectoration, and asthma. Steroidal alkaloids are the major biological active constituents in BFC, whereby imperialine is one of the important steroidal alkaloids. So far, there are no studies reporting the effect of imperialine on COPD. In this study, we investigated the effect of imperialine on pulmonary function and structure and inflammation in a COPD-like rat model which was induced by the combination of exposure to CS and intratracheal administration of LPS. Our data show that imperialine mitigates pulmonary functional and structural impairment and suppressed inflammatory response in a COPD-like rat model by mediating expression of related cytokines in lung tissues of the COPD-like rats, such as IL-1*β*, IL-6, IL-8, TNF-*α*, NF-*κ*B, TGF-*β*1, MMP-9, and TIMP-1.

## 1. Introduction

Chronic obstructive pulmonary disease (COPD) is an epidemic and progressive health problem in the world. COPD is characterized by airflow limitation that is usually associated with an abnormal inflammatory response in the lung to various particles or gases [1]. COPD is the third leading cause of death in the world and affects more than 200 million people worldwide [2–4]. Moreover, report published by the World Bank/World Health Organization predicted that social burden of COPD will rank fifth worldwide in 2020 [2].

The pathological changes in COPD subjects include pulmonary inflammation, oxidative damage, imbalance between protease and antiprotease, endothelial cell dysfunction and apoptosis, proteolysis, and vascular remodeling [5–8]. Bronchitis and emphysema are the two main pathological characteristics of COPD [9, 10], which include emphysematous destruction, oxidative stress [11, 12], and inflammation [13] as well as the airways hyperresponsiveness [14]. Notably, inflammation plays a critical role in COPD. For example, macrophages and neutrophils penetrate the airways and alveoli in the early stage of COPD development. And then, these inflammatory cells increase the levels of a complex cascade of inflammatory mediators, including tumor necrosis factor-*α* (TNF-*α*), interleukin- (IL-) 1*β* (IL-1*β*), IL-6, IL-8, and matrix metalloproteinases (MMPs) [15–17]. In addition, release of elastase and reactive oxygen species secreted by inflammatory cells can degrade extracellular matrix components within alveolar walls and then leads to emphysema [18, 19].

So far, the aetiologies of COPD are not completely clear. What is known is just that smoking, environmental pollution, airway hyperreactivity, age, and genetic predisposition are the risk factors for COPD development. In recent years, however, some studies indicated that cigarette smoke (CS) and bacterial infection are two most common and important risk factors for COPD development [20–22]. Moreover, some literatures reported that the efficient experimental COPD model was successfully established by the combination of exposure to CS and intratracheal administration of lipopolysaccharides (LPS) which is the major component of the outer membrane of Gram-negative bacteria [23–25].

Present COPD therapies mainly focus on reducing symptoms and/or preventing exacerbation by using short- and long-acting bronchodilators, muscarinic antagonists, or combination of the long-acting *β*2-agonist with corticosteroids [26]. Unfortunately, they have limited effect on reducing the progression of COPD or suppressing the inflammatory response in the lung [27]. In addition, the drugs for treatment of COPD have severe side effects to COPD patients [28]. For example, they can induce skin bruising, reduction of bone density, muscle weakness, and respiratory failure [29, 30]. Therefore, safe and effective alternative therapy agents for COPD have long been anticipated [28]. Recently, an increasing number of studies focus on investigation of potential of agents from traditional medicine. One study showed that baicalin, which is an extract from roots of the plant* Scutellaria baicalensis*, inhibits inflammation and NF-*κ*B activation in cigarette smoke induced inflammatory models [31]. Another research reported that Xiaoqinglong decoction could significantly mitigate pulmonary functional and structural impairment in a COPD-like rat model [32].

*Fritillaria cirrhosa* D. Don, which belongs to the family Liliaceae, is primarily distributed in the southwestern China. Bulbs of* F. cirrhosa* (BFC) have been used in many Asian countries for a long time to treat pulmonary diseases, such as cough, expectoration, asthma, and cancer [33]. Some studies indicate that steroidal alkaloids are the major active compositions in BFC [34]. Pharmacological studies suggest that alkaloids from BFC exhibit remarkable antitussive, expectorant, antiasthmatic properties [35, 36], hypotensive effect [37], antibacterial activity, and antitumor effect [33, 38, 39]. Furthermore, some studies showed that imperialine, which is the major alkaloid isolated from BFC [35, 36], is a selective surmountable antagonist at M2 receptors [40] and has the anticholinergic activity [41]. In our previous study, imperialine not only shows significant antitussive and expectorant activities but also exhibits notable anti-inflammatory effect [35, 36].

However, to the best of our knowledge, there are no studies reporting the treatment effect of imperialine on COPD. Therefore, in the present study, we investigated the effect of imperialine on pulmonary function and structure and inflammatory response in the COPD-like rat model which is induced by the combination of exposure to CS and intratracheal administration of LPS. Furthermore, we tried to explore the possible mechanisms of action by evaluation of cytokines level, such as interferon-*γ* (IFN-*γ*), nuclear factor-*κ*B (NF-*κ*B), transforming growth factor-*β*1 (TGF-*β*1), MMP-9, and tissue inhibitor of metalloproteinase-1 (TIMP-1).

## 2. Materials and Methods

### 2.1. Animals and Groups

Wistar rats (280 ± 40 g) were purchased from Experimental Animal Center of West China College of Pharmacy, Sichuan University (Certificate number SCXK (Chuan) 2014-11, Chengdu, China). The rats were housed in laboratory at room temperature (22–24°C) and constant humidity (40–70%) with a 12 h light-dark cycle and provided food and water* ad libitum* [31, 32]. All procedures were in strict accordance with the Chinese legislation on the use and care of laboratory animals and the guidelines established by Institute for Experimental Animals of Sichuan University and were approved by the Sichuan University Committee on Animal Care and Use. After one week of adaptation, eligible animals were randomly assigned into six groups (8 rats in each group) [32].

*Group 1 (control group)*. Rats were exposed to fresh air instead of CS and treated with PBS instead of LPS. They were intragastrically given solvent vehicle solution (1% Tween 80 solution, 1.0 mL/100 g) at 1 h before exposure of fresh air, twice/day. 

*Group 2 (COPD model)*. Rats were exposed to CS and posttreated with LPS. They were intragastrically given solvent vehicle solution (1.0 mL/100 g) at 1 h before exposure of CS, twice/day. 

*Group 3 (the low dose of imperialine)*. Rats were exposed to CS and posttreated with LPS. They were intragastrically given imperialine (3.5 mg/kg) at 1 h before exposure of CS, twice/day. 

*Group 4 (the high dose of imperialine)*. Rats were exposed to CS and posttreated with LPS. They were intragastrically given imperialine (7.0 mg/kg) at 1 h before exposure of CS, twice/day. 

*Group 5 (the positive control)*. Rats were exposed to CS and posttreated with LPS. They were intragastrically given dexamethasone sodium phosphate (DSP, 1.0 mg/kg) at 1 h before exposure of CS, once/3 days.

### 2.2. Establishment of COPD-Like Rat Model

The COPD-like rat model was established by combination of exposure of CS and intratracheal instillation of LPS described as previous studies [25, 31, 32, 42]. A commercially available filter cigarette (Tianxiaxiu brand cigarette, Tobacco Chuanyu Industrial Co. Ltd., China) contains 0.8 mg of nicotine and 10 mg of tar. Different groups of animals were placed into different chambers (dimensions: 60 × 40 × 30 cm^3^, 72 L), respectively. The animals from Groups 2–5 were exposed to the smoke equivalent of 5 cigarettes for 1 h per time, twice/day from day 2 to day 60 (except day 30). The second CS exposure was performed at 4 h after the first exposure. Each cigarette was puffed 15 times for 3 min at the rate of 5 puffs/min. One puff meant drawing 35 mL of CS into a 50 mL syringe and then blowing this CS, which was diluted to 4-5% with air, into the chamber. Fresh air inhalation was performed for 1 min after every 3 min of CS exposure. LPS (1 *μ*g/*μ*L in PBS; 100 *μ*L/rat) was instilled into rats through the trachea after they were anesthetized with 1.5% pentobarbital sodium (50 mg/kg) at day 1 and day 30 [32].

### 2.3. General Appearance Observation and Body Weight Measurement [32]

Animal general appearance was observed during the whole experiment, which included animal movement, fur appearance, weight growth, respiration situation, and cough severity. Animals were weighted from day 1 to day 60. Animal body weight growth index was calculated by the formula: body weight growth = body weight after experiment − body weight before experiment (g).

### 2.4. Pulmonary Function Measurement

The change of pulmonary function, one of the key features of COPD, was measured using the animal sealed unrestrained Whole Body Plethysmograph (Buxco Research System, Wilmington, USA) on day 61 [43, 44]. After induction of anesthesia by intraperitoneal administration of 1.5% pentobarbital sodium (50 mg/kg), the trachea was opened with an inverted T-shaped incision and rapidly intubated and then we connected an endotracheal cannula to the flow transducer. As the animal breathes in and out, the up and down movement of the thorax cage changes the volume of the box. These changes in volume are then converted to electrical signal through a pressure transducer and amplifier and processed by computer and analyzed by the software. Ratios of the forced expiratory volume at 0.3 s and forced vital capacity (FEV_0.3_/FVC), functional residual capacity (FRC), residual volume (RV), forced vital capacity (FVC), dynamic lung compliance (Cdy), tidal volume (TV), peak expiratory flow (PEF), peak inspiratory flow (PIF), and minute volume (MV) were calculated [45, 46].

### 2.5. Preparation of Peripheral Blood, Serum, Bronchoalveolar Lavage Fluid (BALF), Lung Tissue, and Leukocyte Counting

After pulmonary function measurement, the blood was collected, and serum sample was prepared and stored in −80°C for the analysis of inflammatory cytokines. The blood was analyzed using the ABX blood analyzer (JN1212-ABX-MICROS, France). BALF samples were obtained by lavaging left lung lobes with saline for three times (4 mL, 3 mL, and 3 mL) and centrifuged for 10 min (3500 rpm, 4°C). The cell-free supernatants were stored at −80°C for subsequent cytokine analysis, and the sediment cells were resuspended in 0.5 mL PBS for determination of the number of leukocytes using standard morphologic criteria after Wright-Giemsa staining [25, 31, 42]. After preparation of BALF, the middle lobes of the right lungs were then fixed in 4% neutral buffered paraformaldehyde immediately for the histological and immunohistochemical examination.

### 2.6. Cytokine Analysis in Serum

To investigate the effects of imperialine on cytokine in the COPD-like rat model, levels of several cytokines (IL-6, IL-8, TNF-*α*, IL-1*β*, and TGF-*β*1) in serum were measured by enzyme-linked immunosorbent assay (ELISA) using respective kits (CUSABIO, Wuhan, China) according to the manufacturer's instructions.

### 2.7. Histological Examination and Morphological Study

The histological evaluation was performed as described previously [31]. All lung tissues were fixed in 4% neutral buffered paraformaldehyde and processed for paraffin embedding according to standard histological procedures. Every lung tissue was randomly cut into 4 *μ*m thick films and stained with hematoxylin and eosin (H & E). Histological examination was carried out by microscopy (Nikon Eclipse E100). The pathologic changes and scoring for the lung tissues were examined according to the intensity of emphysema and bronchiole stenosis as described previously [47, 48] by three pathologists who were blinded to the group assignments.

To further evaluate pathologic changes of the lung tissues, mean linear intercept (MLI), mean alveolar septal thickness (MAST), and mean alveolar number (MAN) were measured as described previously [45, 49, 50]. Nine different fields of view from every tissue section were photographed at different magnification (MLI: 200x, MAST: 400x, and MAN: 400x) for further analysis. For measurement of MLI, an overlay consisting of horizontal and vertical lines was placed over each field. The number of alveolar intercepts (NI) at the intersection point of the two lines on the overlay was determined. MLI is calculated using the following equation: MLI =* L*/NI (*L* is the total length of two lines on the overlay) [45, 49, 50]. For measurement of MAST, an overlay consisting of horizontal and vertical lines was placed over each field. We counted the number of septa (NS) and measured septal thickness (ST) of the every alveolar at the intersection point of the two lines and calculated the MAST using the following equation: MAST = ST/NS. For measurement of MAN which is an indicator for density of alveoli, we determined the number of alveoli (NA) in each field and measured each field area (*S*) and calculated the MAN using the following equation: MAN = NA/*S*.

### 2.8. Immunohistochemical Examination

The immunohistochemical measurement of NF-*κ*B p65, TGF-*β*1, MMP-9, and TIMP-1 was carried out by standard immunohistochemical techniques [31, 33, 38]. Briefly, every lung tissue was cut into 4 *μ*m of film. The sections were dried overnight at 37°C, subsequently deparaffinized in xylene, and hydrated through a graded series of alcohol. Then, these sections were put into sodium citrate buffer solution (pH = 6.0) at 95°C for 40 min to recover antigen and incubated with 3% H_2_O_2_ for 15 min to inactivate endogenous peroxidase. Nonspecific binding sites were blocked for 1 h in PBS containing 1.5% normal serum. Then, the slides were incubated with primary NF-*κ*B p65 (1 : 200), TGF-*β*1 (1 : 100), or MMP-9 (1 : 100) (Abcam, Shanghai, China) antibody at 4°C overnight and then incubated with HRP-conjugated secondary antibody (Gene Tech Company, Shanghai, China) for 45 min at 37°C. The immune reactions were visualized by immersing the slides in DAB reagent. The slides were counterstained with hematoxylin and then dehydrated with sequential ethanol for sealing. Digital images were obtained from eight representative fields (100x) of each section using a digital camera attached to light microscopy (Nikon Eclipse E100). Immunohistochemically positive staining for NF-*κ*B p65, TGF-*β*1, or MMP-9 in cells showed brown granules and their integrated optical density (IOD) was quantified using Image-Pro plus software (Media Cybernetics Inc., USA) as described [31, 33, 38].

### 2.9. Statistical Analysis

The results are expressed as mean ± SEM. One-way analysis of variance (ANOVA) test was performed for multiple comparisons and Student's *t*-test was carried out for comparison of two groups [11]. All analyses were performed using the SPSS statistics 17.0 software package (LEAD Technologies, Inc., USA). *P* value of less than 0.05 was considered to be statistically significant.

## 3. Results

### 3.1. Animal General Appearance and Weight Changes

**Figure 1:**
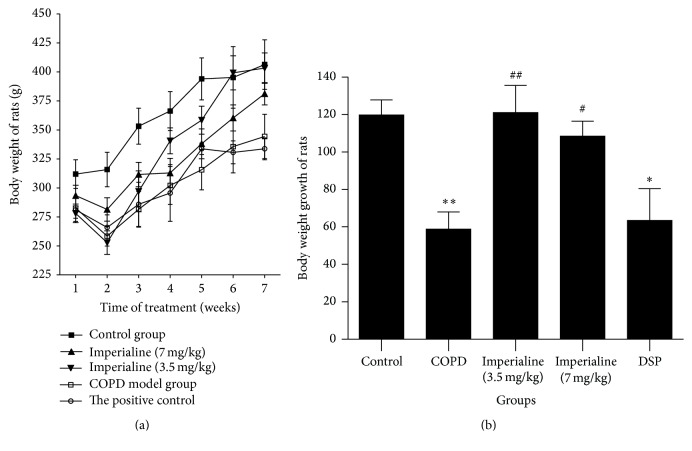
The effect of imperialine on (a) the body weight and (b) body weight growth of rats. Results were expressed as the mean ± SEM (*n* = 10). Significant differences compared with the control group were designated as ^*∗*^ *P* < 0.05 and ^*∗∗*^ *P* < 0.01. Significant differences compared with the COPD group were designated as ^#^ *P* < 0.05 and ^##^ *P* < 0.01.

The rats in the control group were active and restless, with smooth and burnished fur. Their body weight increased gradually (Figure 1(a)) and respiration was stable. The rats in the model group usually stayed still with gathered fur. Their body weight increased slowly and respiration was short accompanied by frequent cough. These symptoms in the therapeutic group were obviously alleviated [32]. In addition, weight growth of rats in the treated groups was significantly higher than that in COPD group (Figure 1(b)).

### 3.2. Pulmonary Function [45, 50]

**Figure 2:**
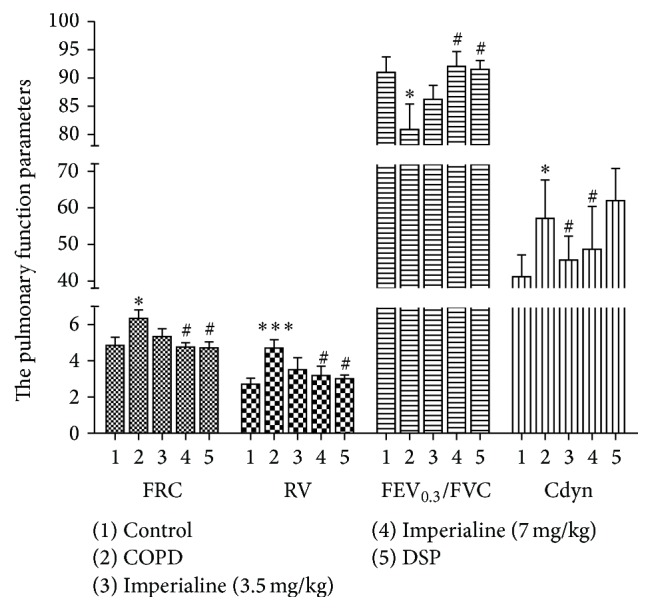
The pulmonary function parameters of rats in different groups. The reported values are the means ± SEM. Significant differences compared to the control group are indicated by ^*∗*^ *P* < 0.05 and ^*∗∗∗*^ *P* < 0.001. Significant difference compared to the COPD model group is indicated by ^#^ *P* < 0.05.

Impairment of pulmonary function is a hallmark of COPD [51]. Therefore, pulmonary function parameters including FEV_0.3_/FVC, FRC, RV, FVC, Cdy, TV, PEF, PIF, and MV were determined. The results of pulmonary function parameters are shown in Figure 2. The values of FRC, RV, and Cdyn of rats in COPD model group were significantly higher than those of rats in control group, while the value of FEV_0.3_/FVC of rats in COPD model group was significantly lower than that of rats in control group. Treatment with imperialine at high dose resulted in a significant decrease of the values of FRC, RV, and Cdyn and increase of the value of FEV_0.3_/FVC as compared with the COPD model group. There are no significant differences in these parameters between the high dose of imperialine group and the control group. The positive control DSP also could improve these values as compared with the COPD model group. In addition, there are no significant changes in FVC, PEF, TV, MV, and PIF of the rats between the five different groups (data not shown).

### 3.3. Leukocyte Counts in BALF

**Figure 3:**
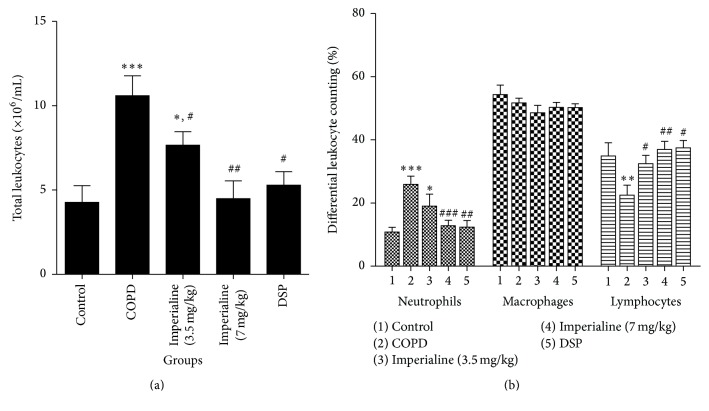
The effect of imperialine on (a) the total cell number of leukocytes and (b) the number of the different leukocytes in BALF. Results were expressed as the mean ± SEM (*n* = 10). Significant differences compared with the control group were designated as ^*∗*^ *P* < 0.05, ^*∗∗*^ *P* < 0.01, and ^*∗∗∗*^ *P* < 0.001. Significant differences compared with the COPD group were designated as ^#^ *P* < 0.05, ^##^ *P* < 0.01, and ^###^ *P* < 0.001.

To determine the effect of imperialine on inflammation in the COPD-like rat model, the total cell number of leukocytes and the number of the different leukocytes in BALF were determined [31, 42]. As shown in Figure 3(a), the total number of leukocytes in the COPD group is the highest in the 5 groups. Imperialine and DSP could significantly decrease the number of total leukocytes in BALF in the COPD-like rat. There are no significant differences between the control group and the high dose of imperialine group or the DSP group.

In Figure 3(b), the percentage of neutrophils in the COPD group is significantly higher than that in control group. Imperialine at high dose and DSP could significantly decrease the percentage of neutrophils in BALF in the COPD-like rats, and there are no significant differences between the control group and the high dose of imperialine group or DSP group. Besides, there is no significant difference in the percentage of macrophages between the five groups. The percentage of lymphocytes in the COPD group is significantly lower than that in the control group and the low and high dose of imperialine and DSP groups. There are no significant differences in percentage of lymphocytes between the control group and the low or high dose of imperialine group or DSP group.

### 3.4. Total and Different Peripheral Blood Cell Counts

**Figure 4:**
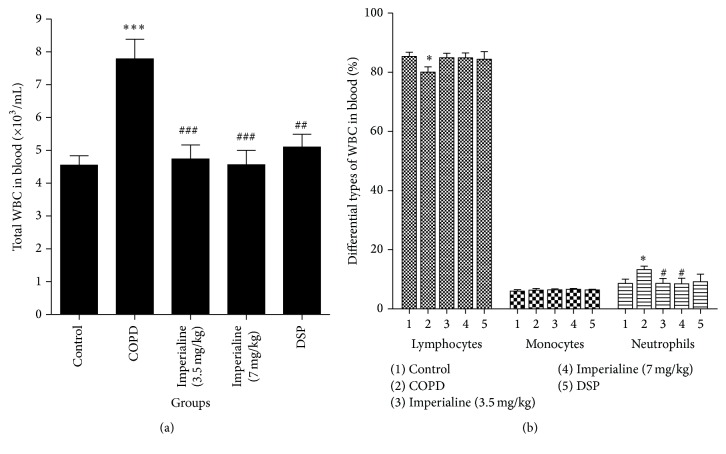
The effect of imperialine on the number of (a) total white blood cells (WBC) and (b) different types of WBC in the blood. Results were expressed as the mean ± SEM (*n* = 10). Significant differences compared with the control group were designated as ^*∗*^ *P* < 0.05 and ^*∗∗∗*^ *P* < 0.001. Significant differences compared with the COPD group were designated as ^#^ *P* < 0.05, ^##^ *P* < 0.01, and ^###^ *P* < 0.001.

We further determined the number of total white blood cells (WBC) and different types of WBC in the blood [28, 48]. In Figure 4(a), the WBC counts in the blood of rats in the COPD group are significantly higher than that in the other four groups. The total WBC number in the treated groups with imperialine or DSP was significantly decreased compared to the COPD group. In Figure 4(b), neutrophils percentage in the blood of rats in the COPD group is significantly higher than that in the control group. Imperialine could decrease the percentage of neutrophils significantly, whereas the positive control DSP does not. Besides, the percentage of lymphocytes in the COPD group is lower than that in the control group. In addition, there is no significant difference in the number of monocytes between different groups.

### 3.5. Inflammatory Cytokines in Serum

**Figure 5:**
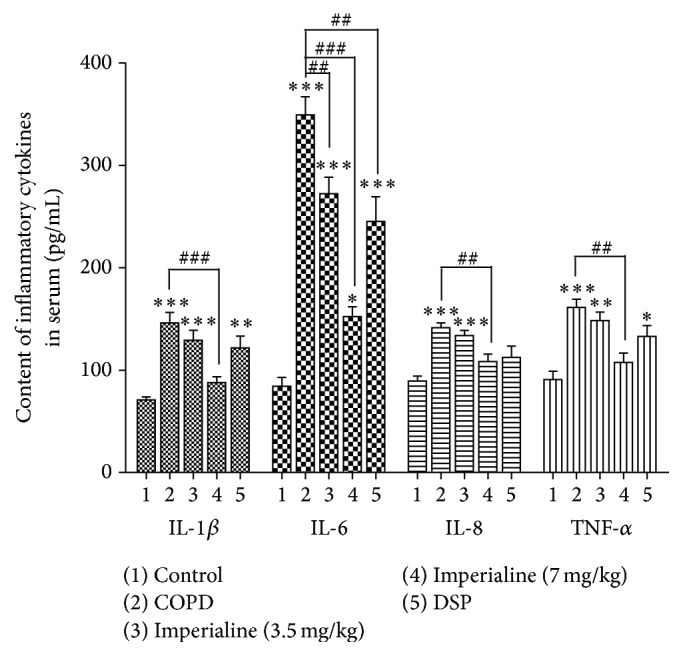
The effect of imperialine on levels of the inflammatory cytokines in serum. Results were expressed as the mean ± SEM (*n* = 10). Significant differences compared with the control group were designated as ^*∗*^ *P* < 0.05, ^*∗∗*^ *P* < 0.01, and ^*∗∗∗*^ *P* < 0.001. Significant differences compared with the COPD group were designated as ^##^ *P* < 0.01 and ^###^ *P* < 0.001.

IL-1*β*, IL-6, IL-8, and TNF-*α*, which are regarded as chemokines of neutrophils and the key inflammatory cytokines of COPD, were determined using ELISA method [31]. As shown in Figure 5, the plasma from the COPD group displays markedly higher levels of the four cytokines than that in the control group, and the high dose of imperialine could significantly decrease the levels of the four cytokines in serum. There are no significant differences in the levels of IL-1*β*, IL-8, and TNF-*α* between the control group and the high dose of imperialine group. The low dose of imperialine and the positive control DSP only could significantly decrease the level of IL-6.

### 3.6. Histopathologic Analysis

**Figure 6:**
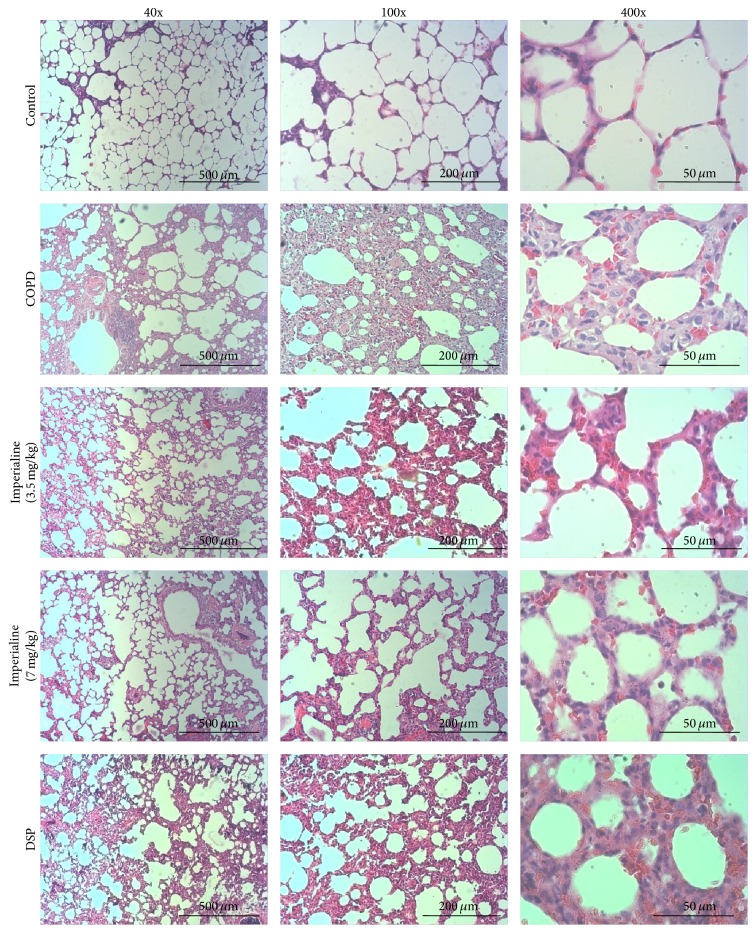
Typical H & E staining images. Histological examination of lung tissues of rats in different groups (magnification: 40x, 100x, and 400x).

**Figure 7:**
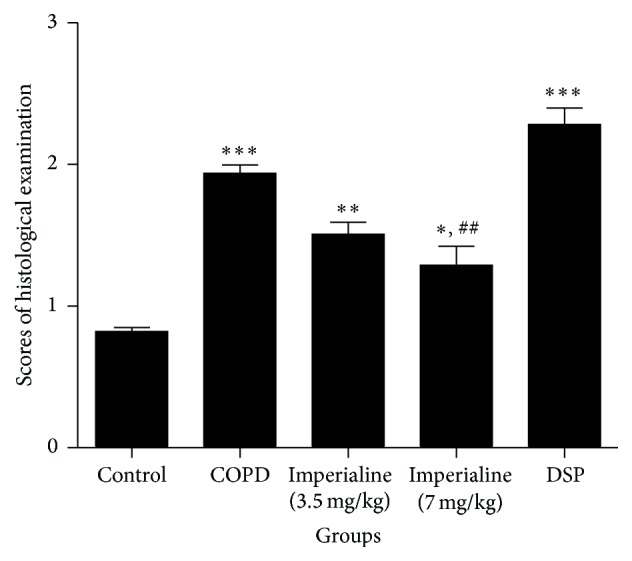
The scores of histological examination of lung tissues of rats in different groups. Results were expressed as the mean ± SEM (*n* = 10). Significant differences compared with the control group were designated as ^*∗*^ *P* < 0.05, ^*∗∗*^ *P* < 0.01, and ^*∗∗∗*^ *P* < 0.001. Significant differences compared with the COPD group were designated as  ^##^ *P* < 0.01.

The tissue sections were stained with H & E and photographed. As shown in Figure 6, the tissues of the rats in the COPD group show significant inflammatory cell infiltration, airway mucosal edema, increase of mucus secretion, localized emphysema, gas cavity stenosis, and other pathological manifestations [43]. Imperialine alleviates the pathological impairment partly. As shown in Figure 7, score of histological examination in the COPD group is significantly higher than that in the control group. In addition, the high dose of imperialine could significantly mitigate the bronchiole stenosis, whereas the low dose of imperialine as well as DSP does not.

**Figure 8:**
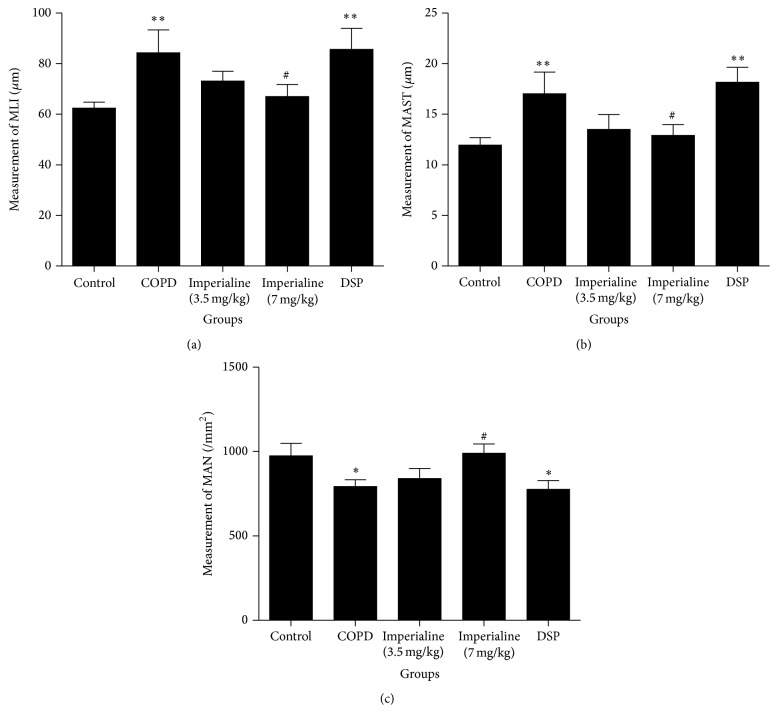
The effect of imperialine on (a) mean linear intercept (MLI), (b) mean alveolar septal thickness (MAST), and (c) mean alveolar number (MAN) of lung tissues of rats in different groups. Results were expressed as the mean ± SEM (*n* = 10). Significant differences compared with the control group were designated as ^*∗*^ *P* < 0.05 and ^*∗∗*^ *P* < 0.01. Significant differences compared with the COPD group were designated as ^#^ *P* < 0.05.

As shown in Figure 8(a), MLI of COPD group is obviously higher than that of the control group. The high dose of imperialine could significantly reduce MLI and there is no significant difference between the high dose of imperialine and control groups. The low dose of imperialine could slightly decrease MLI, whereas DSP does not. As shown in Figure 8(b), MAST of COPD group is significantly higher than that of the control group. The high dose of imperialine could significantly reduce MAST and its value is closest to that of control group. The low dose of imperialine could slightly decrease MAST. As shown in Figure 8(c), the value of MAN of the COPD group is obviously lower than that of the control group. The high dose of imperialine could significantly increase value of MAN. In addition, the low dose of imperialine could slightly increase MAN, whereas DSP does not.

### 3.7. Expression of NF-*κ*B p65, TGF-*β*1, MMP-9, and TIMP-1 in Lung Tissues

**Figure 9:**
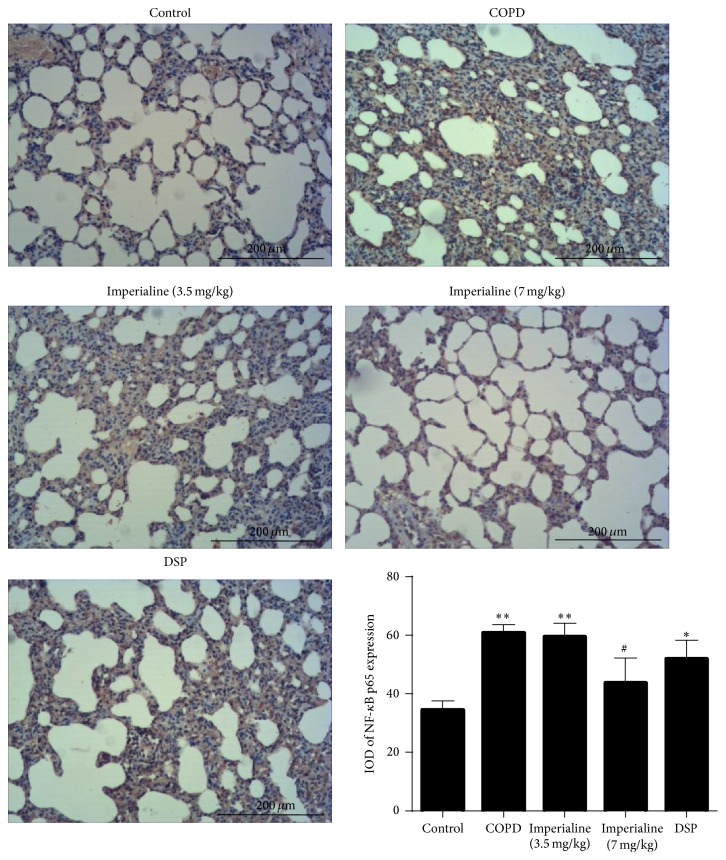
Immunohistochemical staining of NF-*κ*B in lung tissues of rats in different groups and IOD of NF-*κ*B expression. Results were expressed as the mean ± SEM (*n* = 10). Significant differences compared with the control group were designated as ^*∗*^ *P* < 0.05 and ^*∗∗*^ *P* < 0.01. Significant differences compared with the COPD group were designated as ^#^ *P* < 0.05.

The NF-*κ*B p65 immunohistochemical staining and IOD of NF-*κ*B p65 expression are shown in Figure 9. The IOD of NF-*κ*B p65 protein expression in lung tissues in the COPD and low dose of imperialine and DSP groups is significantly higher than that in the control group. The high dose of imperialine significantly decreased NF-*κ*B p65 expression in the COPD-like rats. In addition, DSP could slightly reduce NF-*κ*B p65 expression, but there is no significance.

**Figure 10:**
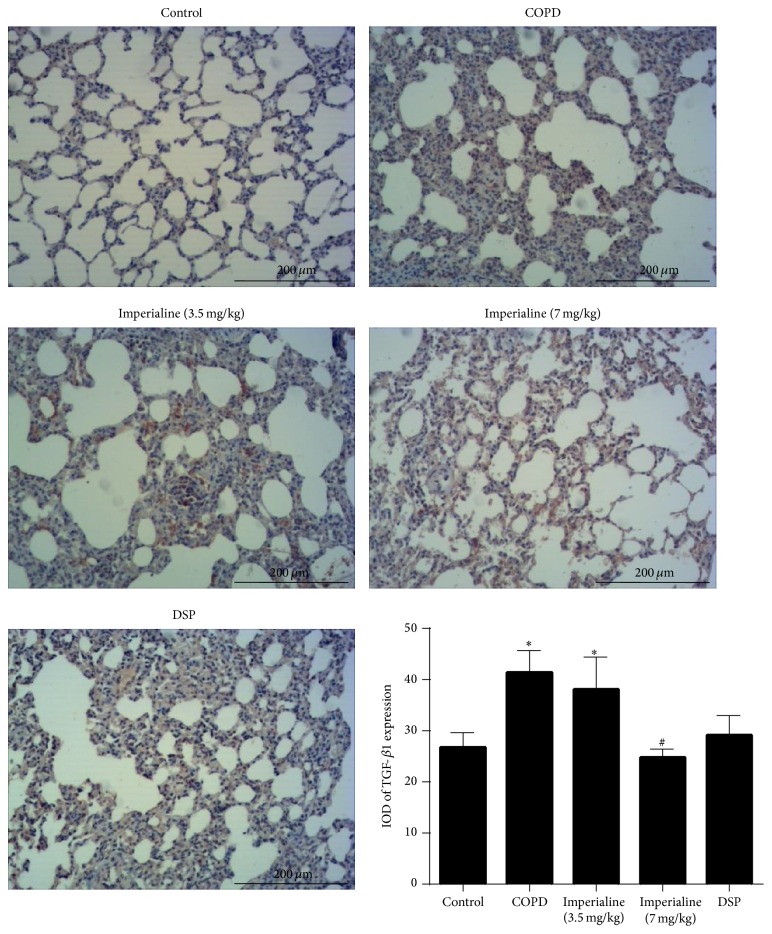
Immunohistochemical staining of TGF-*β*1 in lung tissues of rats in different groups and IOD of TGF-*β*1 expression. Results were expressed as the mean ± SEM (*n* = 10). Significant differences compared with the control group were designated as ^*∗*^ *P* < 0.05. Significant differences compared with the COPD group were designated as ^#^ *P* < 0.05.

Expression of TGF-*β*1 in the tissues from the different groups is shown in Figure 10. The IOD of TGF-*β*1 protein expression in lung tissues in the COPD and low dose of imperialine groups is significantly higher than that in the control group. The high dose of imperialine could decrease TGF-*β*1 protein expression significantly. Additionally, DSP could slightly decrease NF-*κ*B p65 expression in comparison with COPD group, whereas there is no significance.

**Figure 11:**
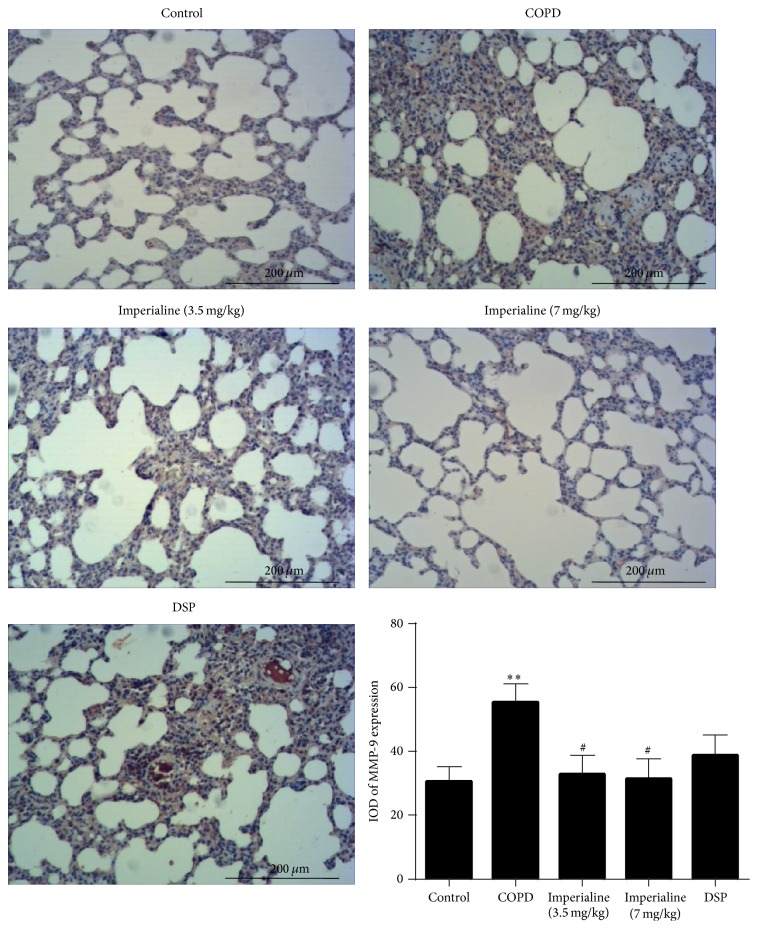
Immunohistochemical staining of MMP-9 in lung tissues of rats in different groups and IOD of MMP-9 expression. Results were expressed as the mean ± SEM (*n* = 10). Significant difference compared with the control group was designated as ^*∗∗*^ *P* < 0.01. Significant difference compared with the COPD group was designated as ^#^ *P* < 0.05.

**Figure 12:**
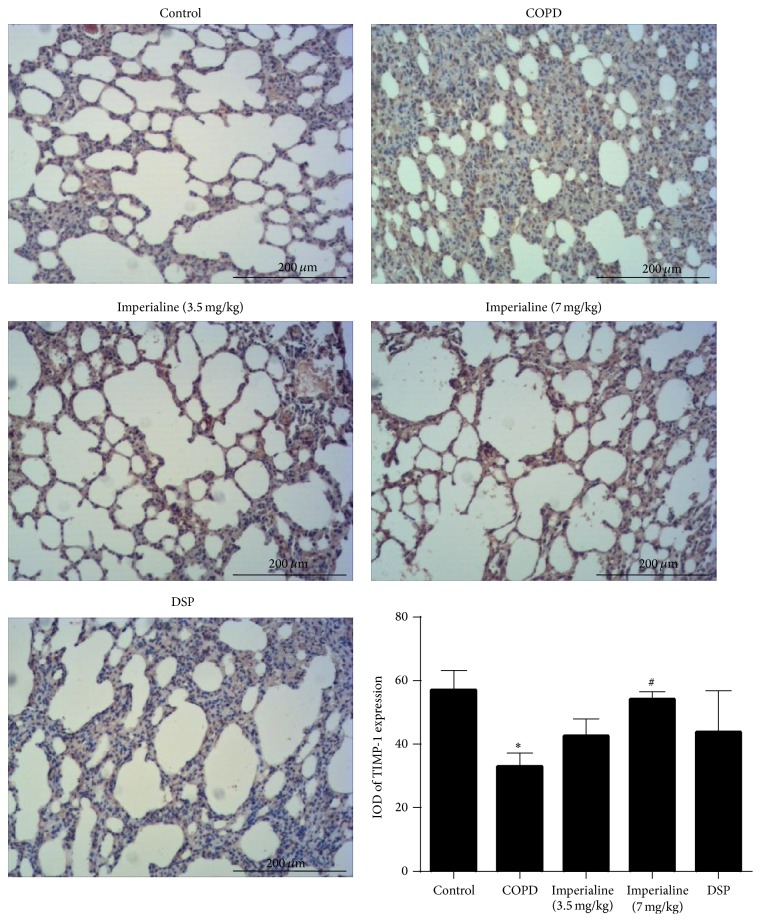
Immunohistochemical staining of TIMP-1 in lung tissues of rats in different groups and IOD of TIMP-1 expression. Results were expressed as the mean ± SEM (*n* = 10). Significant difference compared with the control group was designated as ^*∗*^ *P* < 0.05. Significant difference compared with the COPD group was designated as ^#^ *P* < 0.05.

We also investigated the expression of MMP-9 and TIMP-1 in the lung tissues of rats in the different groups [52]. As shown in Figure 11, the IOD of MMP-9 protein expression in the COPD group is significantly higher than that in the control group. The low and high dose of imperialine could decrease MMP-9 protein expression significantly as compared to the COPD group. Besides, DSP could slightly reduce MMP-9 expression compared with the COPD group, but there is no significance. As shown in Figure 12, TIMP-1 was reduced significantly in the lung tissues of rats in the COPD group. The high dose of imperialine could elevate TIMP-1 protein expression significantly as compared to COPD group. The low dose of imperialine and DSP could slightly increase TIMP-1 expression compared with COPD group, whereas there is no significance.

## 4. Discussion

In this study, we established the COPD-like rat model induced by the combination of exposure of CS and intratracheal instillation of LPS to evaluate the effect of imperialine on pulmonary function and structure and inflammatory response. CS, one of the major pathogeneses of COPD [25], contains high concentration of reactive oxygen species, which could induce chemotactic factors and accumulate neutrophils in the lung [31]. Furthermore, COPD-like rat model was established successfully by repeated exposure to CS [45, 53]. Another most common cause of COPD is viral and bacterial infections [23]. A recent study indicated that 78% of COPD patients were subjected to respiratory viral and/or bacterial infections [54]. Specifically, the Gram-negative bacteria are the most commonly isolated bacterial pathogens from COPD patients [55]. In recent several years, more efficient COPD-like models induced by the combination of CS plus LPS were reported [23–25], which was characterized by chronic lung inflammation, emphysema, elevation of airway resistance, and so on. In this study, the rats in the COPD group showed low weight growth, pulmonary functional and structural impairment, bronchiole stenosis, accumulation of neutrophils, induction of inflammatory cytokines in BALF and blood, increase of linear intercept and alveolar septal thickness, decrease of alveolar number, increase of the inflammatory cytokines expression in the lung, and imbalance between MMP-9 and TIMP-1 in the lung, which clearly suggest an animal model of COPD.

Numerous clinical studies have shown that COPD patients developed skeletal muscle atrophy and body weight loss [43]. The slowdown in body weight growth, an indicator of deteriorated nutrition, has been found to increase the hospitalization rate in patients with COPD [56], the rate of mechanical ventilation necessary, and mortality [57]. In addition, the survival time of patients with COPD deterioration is highly related to the body weight growth. In this study, we found that weight growth of rats in the COPD group is significantly lower than that in the control group, and imperialine significantly elevated the weight growth.

The decline of pulmonary function is a key hallmark in diagnosing COPD. FEV/FVC ratio is an indicator of lung injury in obstructive lung disease, and FRC and RV are expected for the presence of emphysema [45, 51, 57]. In this study, we tested the FRC, RV, FEV_0.3_/FVC, FVC, PEF, TV, MV, Cdyn, and PIF of rats. The results showed that there is a significant increase in FRC and RV concomitantly with the obvious decrease in FEV_0.3_/FVC of rats in the COPD group as compared to the control group, which is consistent with clinical observation in patients with COPD [45, 50]. Treatment with imperialine partly but significantly reversed the pulmonary function decline and ameliorated airflow obstruction in the COPD-like rats. Meanwhile, it could decrease the value of FRC and RV, suggesting that intervention of imperialine could attenuate emphysema in COPD-like rats.

Abnormal lung inflammation plays a critical role in the onset and progression of COPD [42]. The previous studies reported that both CS and LPS directly damaged airway epithelium and activated macrophages and lymphocytes to generate proinflammatory cytokines (such as TNF-*α*, IL-6, IL-8, and IL-1*β*), which then activated neutrophils, leading to chronic bronchial inflammation and emphysema [31, 42]. In this study, the total cell number of leukocytes and percentage of neutrophils in BALF and peripheral blood from COPD group are significantly higher as compared to the control group. Imperialine significantly reduced the total number of leukocytes and percentage of neutrophils in BALF and peripheral blood, whereas DSP does not. However, the number of lymphocytes was decreased in BALF and peripheral blood in response to stimuli of LPS and CS, which might be due to the migration of lymphocytes into the interstitium of the lung [58]. In addition, IL-8 is a critical protein factor in the recruitment of leukocytes to sites of inflammation. IL-6, TNF-*α*, and IL-1*β* are the critical inflammatory factors in human COPD [31, 59]. In this study, CS exposure and intratracheal instillation of LPS resulted in increasing levels of IL-8, IL-6, TNF-*α*, and IL-1*β* in serum of rats, which was effectively inhibited by imperialine. The results indicated that imperialine displayed anti-inflammatory effect. In fact, previous studies also showed anti-inflammatory effects of bulbs of* F. cirrhosa* and its constituents, which support the results in the present study [35, 36].

The histological changes in lung tissues of rats in the COPD group include enlargement of lung air space, formation of pulmonary bullae, small airway remodeling, and destruction of septal walls of alveoli [45], which indicated that the COPD-like rat model induced by combination of exposure of CS and intratracheal instillation of LPS was developed successfully. Treatment with imperialine alleviated morphological impairments in the COPD-like rats, suggesting that it may slow down the progression of COPD. Since imperialine significantly reduced the inflammatory response in the lung by decreasing the number of leukocytes, percentage of neutrophils, and the levels of proinflammatory mediator, we speculate that its effect on the pathological changes is due to ameliorating inflammatory response.

In addition, MLI, MAST, and MAN in the different groups were measured to evaluate their distal air space size, alveolar septal thickness, and alveolar density [45]. MLI and MAN are the accurate and efficient stereological approach for the direct and unbiased quantitative analysis of lung structure [60, 61]. The results provided a direct evidence of lung injury in the COPD-like rats, which is consistent with the histopathological changes in lung tissues. MAST reflects the degree of small airway remodeling; the results in this study show that imperialine also could influence the remodeling of small airway [45].

NF-*κ*B is a critical signaling molecule in inflammation of COPD [31]. In this study, exposure of CS and intratracheal instillation of LPS increased the levels of NF-*κ*B-dependent proinflammatory mediators such as TNF-*α*, IL-6, IL-8, and IL-1*β* in blood. Therefore, we investigated NF-*κ*B p65 expression using immunohistochemical staining method. Our data indicated that imperialine decreased NF-*κ*B p65 expression significantly, suggesting that imperialine inhibited production of chemotactic cytokines by inhibiting activation of the transcription factor NF-*κ*B.

One of the characteristics of COPD is the airway wall thickness accompanied by an increase of smooth muscle mass and the deposition of extracellular matrix [62–65]. TGF-*β*1 is a multifunctional growth factor that can modulate cellular proliferation and differentiation and induce synthesis of extracellular matrix proteins including collagens and fibronectin [62]. Previous research indicated that there was a significant increase of TGF-*β*1 in airway epithelial cells in the subjects with COPD [62, 66]. In this study the immunohistochemical results also showed that expression of TGF-*β*1 in the lung tissues from the COPD group significantly increased as compared with the control group, whereas imperialine could decrease TGF-*β*1 protein expression significantly as compared to the COPD group.

An imbalance of protease-antiprotease is a key perpetuating factor in tissue remodeling and development of emphysema. TIMP/MMP imbalance can lead to pulmonary emphysema [52, 67]. MMP-9 is believed to be the critical enzyme involved in the degradation of the extracellular matrix components during fibrosis and repair processes [52, 68]. Furthermore, MMP-9 can cause airway fibrosis changes and contribute to the COPD development [52]. A significant increase of MMP-9 has been found in BALF samples from patients with COPD compared with non-COPD individuals [69]. This is in agreement with our findings that the level of MMP-9 in the lung tissues of rats in the COPD group was increased. In addition, TIMP-1 can inhibit MMP activity [67]. The previous study reported that level of TIMP-1 was reduced significantly in the lung tissues of rats in the COPD group. Imperialine could decrease MMP-9 protein expression and elevate TIMP-1 protein expression significantly as compared with COPD group. These results indicated that redressing the TIMP-1/MMP-9 imbalance would be one of the mechanisms underlying the protection of imperialine against emphysema.

The positive control DSP could regulate lung function of COPD-like rats and decrease the number of total leukocytes in BALF significantly; however, it could not decrease inflammatory cytokines in serum, alleviate pathologic changes, and reduce the expression of inflammatory cytokines in the lungs, which are consistent with previous reports [47, 51–53]. The study has the limitation that we used a preventive experimental design by treatment of rats with imperialine at the same time as CS exposure. Accordingly, effect of imperialine on developed structural changes of lung in COPD rats remains unknown.

## 5. Conclusion

In conclusion, our data indicated that imperialine mitigated pulmonary functional and structural impairment and suppressed inflammatory response in the COPD-like rat model by mediating expression of related inflammatory cytokines (IL-1*β*, IL-6, IL-8, TNF-*α*, NF-*κ*B, and TGF-*β*1) and redressing the TIMP-1/MMP-9 imbalance in the lung tissues of the COPD-like rats.

## Abbreviations

COPD: Chronic obstructive pulmonary disease
BFC: Bulbs of* Fritillaria cirrhosa* D. Don
CS: Cigarette smoke
TNF-*α*: Tumor necrosis factor-*α*
IL: Interleukin
MMP: Matrix metalloproteinase
LPS: Lipopolysaccharides
IFN-*γ*: Interferon-*γ*
NF-*κ*B: Nuclear factor-*κ*B
TGF-*β*1: Transforming growth factor-*β*1
TIMP-1: Tissue inhibitor of metalloproteinase-1
DSP: Dexamethasone sodium phosphate
FEV_0.3_: Forced expiratory volume at 0.3 s
FVC: Forced vital capacity
FRC: Functional residual capacity
RV: Residual volume
Cdy: Dynamic lung compliance
TV: Tidal volume
PEF: Peak expiratory flow
PIF: Peak inspiratory flow
MV: Minute volume
BALF: Bronchoalveolar lavage fluid
H & E: Hematoxylin and eosin
MLI: Mean linear intercept
MAST: Mean alveolar septal thickness
MAN: Mean alveolar number
NI: Number of alveolar intercepts
NS: Number of septa
ST: Septal thickness
NA: Number of alveoli.
